# Biofloc technology and immunological resilience in pacific white shrimp (*Litopenaeus vannamei*): A mechanistic review

**DOI:** 10.1016/j.cirep.2026.200272

**Published:** 2026-02-16

**Authors:** Yusuf Jibril Habib, Shengkang Li, Mayada Alhoshy, Islam I. Teiba, Xianyuan Zheng, Mohammed F. El Basuini, Akram Ismael Shehata

**Affiliations:** a Department of Medical Analysis, Faculty of Applied Science, Tishk International University, Erbil, Iraq; b Guangdong Provincial Key Laboratory of Marine Biology, Shantou University, Shantou 515063, China; c Institute of Marine Sciences, Shantou University, Shantou 515063, China; d Independent Researcher, Alexandria, Egypt; e Faculty of Agriculture, Tanta University, Tanta 31527, Egypt; f Faculty of Desert Agriculture, King Salman International University, South Sinai, Sinai, Egypt; g Department of Animal and Fish Production, Faculty of Agriculture (Saba Basha), Alexandria University, Alexandria 21531, Egypt

**Keywords:** Biofloc technology, *Litopenaeus vannamei*, Shrimp immunity, Innate immune response, Aquaculture sustainability

## Abstract

Biofloc Technology (BFT) is recognized as an ecologically sustainable aquaculture method that improves water quality, enhances nutrient recycling, and increases disease resilience in cultured crustaceans, especially *Litopenaeus vannamei*. Initially designed to mitigate environmental impact and decrease reliance on water exchange, recent findings suggest that biofloc systems have significant immunomodulatory effects, driven by intricate interactions among host, microbes, and the environment. This review consolidates existing mechanistic insights regarding the influence of BFT on immune function in shrimps, focusing on microbiota modulation, innate immune activation, antioxidant enhancement, and exposure to bioactive microbial metabolites. Biofloc systems promote stable and diverse microbial communities that effectively exclude pathogens, enhance beneficial taxa, and provide immunostimulatory microbial-associated molecular patterns (MAMPs) that activate hemocyte and humoral responses. Furthermore, enhanced water quality and nutrient-dense microbial biomass jointly mitigate oxidative stress, promote physiological homeostasis, and bolster resistance to bacterial and viral pathogens, such as *Vibrio* spp. and white spot syndrome virus. Comparative analyses of biofloc and conventional systems demonstrate the enhanced immunological, nutritional, and environmental efficacy of BFT. However, they also identify significant limitations, such as inconsistent outcomes stemming from variable system management, insufficient long-term immune data, and a limited comprehension of the molecular pathways that regulate host responses. To address these knowledge gaps, standardized immune markers, integrated multiomics analyses, and farm-scale validation are necessary. This review presents a mechanistic framework for enhancing BFT as an immune-focused, climate-resilient approach to sustainable crustacean aquaculture.

## Highlights

• Biofloc systems enhance immunological resilience in *Litopenaeus vannamei* through microbiota modulation, innate immune activation, and antioxidant strengthening.
• Microbial metabolites and MAMPs within biofloc stimulate hemocyte activity, AMP expression, and pathogen surveillance mechanisms.
• Improved water quality and nutrient-rich microbial biomass reduce physiological stress and support stable immune homeostasis.
• Variability in BFT performance underscores the need for standardized immune markers, molecular characterization, and long-term monitoring.
• Future directions include multi-omics integration, immune-targeted probiotics, biofloc–vaccine synergies, and climate-resilient BFT system innovation.

## Introduction

Aquaculture of crustaceans, especially whiteleg shrimp (*Litopenaeus vannamei*) and giant-tiger shrimp (*Penaeus monodon*), has grown quickly in the last few decades to supply the world's seafood needs and generate jobs in many coastal areas. But this increase comes with more disease pressure and environmental problems in intensive culture systems, which means that the business needs to focus on finding long-term ways to control health [1,2].

Outbreaks of disease continue to pose major threats to the sustainability of crustacean farming. Opportunistic bacterial pathogens, particularly *Vibrio* spp., along with viral agents such as White Spot Syndrome Virus (WSSV) and emerging syndromes like Acute Hepatopancreatic Necrosis Disease (AHPND), have led to significant mortalities and economic losses on a global scale [3]. These pathogens take advantage of intensive rearing conditions, high stocking density, and suboptimal water quality, resulting in swift production failures when preventive measures are inadequate [4].

In the past, antibiotics and, more recently, probiotics have been employed to keep infections in aquaculture under control. But using antibiotics raises worries about residue, antimicrobial resistance, and regulatory limits. At the same time, probiotic efficacy can vary depending on the system and the environment. As a result, there is a lot of interest in integrated, ecologically based management strategies that lower the pressure from pathogens while boosting the host's ability to fight off disease [5].

**Fig. 1:**
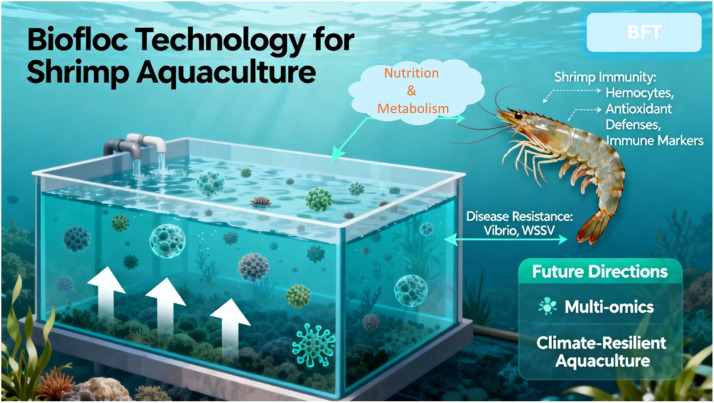
Biofloc Technology (BFT) effect on shrimp wellbeing.

Biofloc Technology (BFT) represents an innovative and sustainable method that enhances water quality, recycles waste nutrients into microbial biomass, and offers a consistent in situ supply of nutrient-rich microorganisms [6,7]. In addition to their nutritional advantages, a growing body of evidence suggests that biofloc systems have the capacity to influence host-associated microbiota, activate innate immune responses, boost antioxidant defenses, and ultimately improve disease resistance in cultured crustaceans, as shown in Fig. 1. The multifunctional effects of BFT position it as a compelling approach for bolstering immunological resilience in intensive shrimp and other crustacean productions [8].

This review seeks to integrate mechanistic insights concerning the effects of biofloc systems on crustacean health via three principal mechanisms: (1) Modification of microbial community structure in both the culture environment and the host gut, (2) Provision of bioactive metabolites and nutritional components that affect immunity and oxidative status, and (3) Improvement of overall water quality and decrease of pathogen load. The assessment will focus on the strength of existing evidence, identify gaps, particularly in molecular and multi-omics domains, and suggest research priorities for translating mechanistic insights into effective field practices. Although this review focuses on *L. vannamei*, this species is a primary experimental paradigm for crustacean immunology in intensive aquaculture due to the abundance of available genetic, cellular, and physiological data. Importantly, several of the immunological processes discussed here, such as hemocytes-mediated phagocytosis, the prophenoloxidase activation system, antimicrobial peptide synthesis, and antioxidant defenses, are substantially conserved among decapod crustaceans.

### Literature search strategy

The literature contained in this narrative review was discovered by extensive searches of major scientific databases such as Web of Science, Scopus, PubMed, and Google Scholar. Searches were conducted using combinations of relevant keywords and Boolean operators, such as "biofloc technology", "*L. vannamei*", "crustacean immunity", "shrimp immune response", "immunological resilience", "host-microbe interaction", "hepatopancreas", "oxidative stress", and "innate immune mechanisms". The emphasis was on peer-reviewed articles published predominantly in the previous twenty years; however, significant earlier studies were included when they were mechanistically pertinent. Titles and abstracts were examined for relevance, and complete texts were assessed to identify publications that offered experimental or mechanistic insights into biofloc systems, immunological regulation, microbial interactions, and physiological responses in *L. vannamei*. Emphasis was placed on studies that reported immunological parameters such as hemocytes activity, immune-related gene expression, antioxidant enzymes, and inflammatory markers, along with responses from the hepatopancreas and microbial communities. The key articles' reference lists were then scrutinized to locate more pertinent publications. The chosen literature was qualitatively synthesized to establish a comprehensive mechanistic understanding of the impact of biofloc technology on the immunological robustness of crustacean shrimps.

## Fundamentals of Biofloc Technology (BFT)

### Principle and microbial ecology of biofloc technology

Biofloc Technology (BFT) is a sustainable aquaculture management strategy that converts nitrogenous waste into microbial biomass in situ, thereby maintaining water quality. By encouraging the growth of beneficial bacteria, BFT converts harmful ammonia and nitrite into biofloc particles that serve as bio-remediators as well as an additional natural food supply for cultivated species [9]. In this system, "biofloc" refers to suspended microbial aggregates formed by microorganisms and organic matter, whereas BFT symbolizes a larger operational strategy that balances nutrient dynamics and microbial activities to sustain intense aquaculture production [8,10].

When a carbon source such as molasses, tapioca, or glycerol is supplied, heterotrophic bacteria require both carbon and nitrogen, which they can obtain from fish and shrimp excrement and uneaten feed. By preventing ammonia and nitrite from circulating, this process reduces toxicity and maintains optimal water quality [11]. The technology encourages effective fertilizer use and better environmental control in intensely cultured settings by constantly immobilizing and recycling nitrogen [12,13].

Biofloc systems are more than just heterotrophic bacteria; they are also complex microbial ecosystems. Bioflocs are made up of autotrophic nitrifying bacteria, microalgae, protozoa, detritus, and organic particles that are held together by extracellular polymeric compounds and exopolysaccharides [14]. Gradually, these microbial assemblages establish a balanced, self-sustaining bio-ecosystem that purifies the water and nourishes cultivated species such as *Oreochromis niloticus* and *L. vannamei* [11,12].

### Nutritional and functional composition of biofloc

Bioflocs contain high levels of microbial protein, essential amino acids, lipids, vitamins, minerals, and bioactive compounds, establishing them as a significant supplemental feed source for aquatic animals. The proximate composition of biofloc biomass generally consists of 25–50% crude protein, 0.5–5% lipid, and a notable ash fraction, which varies based on the carbon source and the microbial community present [15]. Biofloc particles have many vital functions, one of which is to provide extra nutrients. Microbial biomass has proteins and important fatty acids, such as polyunsaturated fatty acids, which can help with growth and the efficiency of converting feed into energy [16]. Secondly, microbial cell wall components, including lipopolysaccharides and peptidoglycans, function as microbial-associated molecular patterns (MAMPs) that activate innate immune responses. These compounds stimulate immune pathways such as phenoloxidase, lysozyme, and phagocytic activity in crustaceans [11,17].

The effects of antagonists and probiotics were investigated. It revealed that certain bacteria associated with biofloc generate antimicrobial compounds that suppress opportunistic pathogens like *Vibrio harveyi* and *V. parahaemolyticus*, thereby lowering the likelihood of disease outbreaks [8,18]. Additionally, bioflocs contribute to the improvement of digestive enzymes. The use of bioflocs has been shown to enhance the activities of digestive enzymes such as amylase, protease, and lipase in fish and prawns, thereby improving feed efficiency and promoting gut health [17,19,20]. The nutritional composition of bioflocs is variable and largely influenced by system-specific conditions, including the type of carbon source used, the C:N ratio, stocking density, and water salinity [16].

### System parameters and operational considerations

The most important operational and design factor influencing BFT performance is the C:N ratio. It determines whether heterotrophic or autotrophic microbial activity is more common. For effective ammonia absorption, a ratio of 15:1 to 20:1 is usually suggested [8,17]. Carbon sources such as molasses or rice bran are commonly used to adjust this ratio. Secondly, BFT systems necessitate vigorous aeration and mixing to ensure the suspension of floc and to sustain elevated dissolved oxygen levels exceeding 5 mg/L. Effective aeration inhibits floc sedimentation and the development of anoxic conditions [14]. Thirdly, BFT systems need competent management. In most shrimp systems, the total suspended solids are kept between 300 and 500 mg/L. Too many solids can make it harder for fish to obtain oxygen and irritate their gills. This is why settling chambers or sludge removal systems are sometimes established [15]. BFT systems also need high water quality parameters. The best values for microbiological and animal performance are pH (7.0–8.0), alkalinity (>100 mg/L as CaCO₃), and temperature (25–32°C). To avoid hazardous build-up, you need to check the levels of ammonia, nitrite, and nitrate on a regular basis [18].

Lastly, biofloc systems can handle high stocking densities, often more than 300–500 shrimp/m³, because they improve water quality and have self-regulating microbial loops [16]. Biofloc technology is an aquaculture management system that relies on microbial communities to improve water quality, recycle nutrients, and support host physiological and immune functions. Successful implementation requires precise regulation of key system parameters, including the carbon-to-nitrogen ratio, aeration, and suspended solids, as well as a mechanistic understanding of the interactions between microbial consortia and the nutritional value of biofloc biomass. The method not only has less of an impact on the environment, but it also makes cultured animals' immune systems stronger and their growth better.

## Overview of crustacean immune system

Crustaceans, including penaeid shrimp and other commercially important aquatic species, lack the classical adaptive immune components found in vertebrates, such as immunoglobulins and antigen-specific lymphocyte-based memory. Their defense against pathogens is therefore primarily mediated by innate immune mechanisms. However, increasing evidence indicates that crustaceans can exhibit innate immune priming and memory-like responses, resulting in enhanced protection upon repeated exposure to pathogens or immunostimulants [21,22]. Their immune system relies on a combination of cellular and humoral defense mechanisms to fight off a wide range of pathogenic organisms, such as bacteria, viruses, fungi, and parasites.

Hemocytes are the main immune cells of crustaceans. They are usually divided into three types: hyaline, semi-granular, and granular. Each type helps with phagocytosis, encapsulation, nodulation, and the release of immune effectors [23]. A wide range of pattern recognition receptors (PRRs), such as β-glucan-binding proteins, lipopolysaccharide-binding proteins (LBPs), peptidoglycan recognition proteins (PGRPs), lectins, and fibrinogen-related proteins, are responsible for humoral immunity. These proteins recognize pathogen-associated molecular patterns and initiate immune cascades [24]. The prophenoloxidase (proPO) activation system is one of the main defense mechanisms in prawns. It catalyzes melanin formation, which immobilizes pathogens and generates cytotoxic reactive intermediates, and produces reactive intermediates that are harmful to invasive microbes [25]. Concurrently, a variety of antimicrobial peptides (AMPs) with wide antibacterial, antifungal, and antiviral properties are expressed by crustaceans, such as penaeidins, crustins, anti-lipopolysaccharide factors, and lysozymes [26]. In *L. vannamei* and other decapods, shared innate immune signaling pathways, like Toll, IMD, JAK/STAT, and MAPK, have been identified at the molecular level. These pathways control AMP expression, apoptosis, and antiviral responses [27]. Transcriptomic and functional studies indicate that viral and bacterial challenges rapidly regulate genes linked with RNA interference, oxidative stress management, and hemocyte death, highlighting the complexity of immune modulation in shrimp [28]. These investigations collectively demonstrate that crustacean immunity constitutes a highly integrated, multi-faceted defense system that responds to environmental variables and microbial exposure.

### Cellular and humoral immunity

The cellular arm is predominantly mediated by hemocytes, which are circulating immune cells present in the hemolymph. Hemocytes are involved in phagocytosis, which is the engulfment and digestion of pathogens; encapsulation, which is the surrounding of large pathogens or foreign material; nodulation, which is hemocyte aggregation around invading microbes; and melanization, which is the generation of melanin to immobilize and kill pathogens via the prophenoloxidase cascade [29,30]. Hemocytes are divided into three types: granular, semi-granular, and hyaline cells, each with a unique role in immune defense [31].

Humoral defense encompasses soluble immune molecules found in hemolymph and tissues. Key immune effectors comprise antimicrobial peptides, including crustins and penaeidins, as well as anti-lipopolysaccharide factors [32,33]. Prophenoloxidase system enzymes are involved in melanization and the destruction of pathogens [25]. Lectins and pattern recognition receptors identify pathogen-associated molecular patterns [34]. Lysozymes degrade bacterial peptidoglycan [35]. Agglutinins and clotting proteins serve to immobilize pathogens and facilitate wound healing [36]. Similarly, bioactive microbial environments and nutritional immunostimulants have been shown to enhance hemocytes activity, phenoloxidase responses, and expression of immune-related genes in freshwater prawns (*Macrobrachium rosenbergii*), crayfish (*Procambarus clarkii*), and other penaeid shrimp (*Penaeus monodon* and *Fenneropenaeus chinensis*). These similarities lend credence to the mechanisms found in *L. vannamei* being more broadly applicable to crustacean aquaculture in general [37,38].

### Oxidative defense mechanisms and it’s significance in aquaculture

Among the key functional components of the crustacean innate immune system, oxidative defense mechanisms play a critical role in neutralizing reactive oxygen species (ROS) generated during pathogen challenge and maintaining cellular homeostasis. During an infection, crustacean immune cells produce reactive oxygen species as part of their respiratory burst activity to destroy invading organisms [39]. Enzymatic antioxidants such as superoxide dismutase, catalase, and glutathione peroxidase keep the oxidative balance and prevent cell damage [40].

Intensive aquaculture subjects’ crustaceans to various environmental stresses, pathogens, and variable water quality, highlighting the importance of immune function as a key factor in the success of farming operations. Instances of the WSSV, AHPND, and Vibrio infections underscore the importance of comprehending immunity and improving host defense mechanisms [41,42]. Consequently, approaches such as biofloc systems, immunostimulants, probiotics, and enhanced nutrition are being examined to influence and bolster innate immunity [8,43].

## Mechanisms of immunological enhancement by biofloc in shrimp

Biofloc technology improves shrimp health in a number of partially related ways. According to empirical research, when penaeid prawns, particularly *L. vannamei* are raised in well-managed biofloc systems, their innate immunological parameters, antioxidant defenses, and challenges to survival all improve. The following is a summary of the main mechanistic pathways and common immune markers in shrimp and their relevance in host-defense studies are summarized in Table 1.

**Table 1:** Common immune markers in shrimp and their relevance in host-defense studies.

Immune Marker	Immune Role	Common Assay	Immune Stimulation	Application in Shrimp Immunity Research	Refs.
Total Hemocyte Count (THC)	Cellular immunity, phagocytosis reservoir	Hemocytometer/flow cytometry	↑ THC = enhanced cellular immune defense	Primary indicator of innate immune status	[45,46]
Differential Hemocyte Count (DHC)	Functional classes of hemocytes (granular, semi-granular, hyaline)	Giemsa stain + microscopy	↑ Granular cells = activation of immune defense	Tracks cellular immune response patterns	[29]
Phenoloxidase (PO) Activity	Melanization, pathogen encapsulation	L-DOPA spectrophotometric assay	↑ PO = strong humoral immunity	Key in crustacean innate immunity and pathogen defense	[23,73]
Pro-Phenoloxidase (proPO)	Precursor for PO, pathogen recognition cascade	ELISA/RT-qPCR/enzyme assay	↑ proPO expression signals immune activation	Central cascade in shrimp defense	[74]
Lysozyme Activity	Bacterial cell-wall lysis	Turbidity reduction/lysoplate	↑ Lysozyme = enhanced antibacterial defense	Major antimicrobial protein	[75]
Respiratory Burst / ROS production	Oxidative killing of microbes	NBT reduction assay	↑ ROS = oxidative immune response	Marker of hemocyte activity	[76]
SOD Activity	Converts superoxide radicals → H₂O₂	Spectrophotometric enzyme assay	↑ SOD = antioxidant protection from immune activation stress	Key antioxidant immune enzyme	[77,78]
Catalase (CAT)	Detoxifies H₂O₂ → H₂O + O₂	Spectrophotometry	↑ CAT = oxidative stress defense	Antioxidant response indicator	[48]
Glutathione Peroxidase (GPx)	Reduces lipid peroxides	ELISA/colorimetric assay	↑ GPx = higher antioxidant immune status	Stress-related immune enzyme	[78,79]
Antimicrobial Peptides (AMPs) *(Penaeidins, Crustins, ALFs)*	Direct antimicrobial activity	RT-qPCR	↑ AMP gene expression = enhanced pathogen defens	Primary humoral antimicrobial response	[80,81]
Lectins / Pattern Recognition Receptors (PRRs)	Recognize microbial PAMPs	ELISA/RT-qPCR	↑ Lectins = improved pathogen recognition	Key pattern recognition molecules	[82]
Nitric Oxide (NO)	Reactive nitrogen defense	Griess assay	↑ NO = enhanced immune response	Involved in pathogen killing	[65,83]
Total Hemolymph Protein	General immune protein reserves	Bradford/biuret	↑ Total protein = immune activation or stress response	General immune health marker	[35]
Survival Rate after Challenge	Global immune protection	Pathogen challenge test	Higher survival = effective immune enhancement	Functional confirmation of immune enhancement	[84]

### Microbiota modulation and colonization resistance

Biofloc systems grow many different types of microorganisms, such as bacteria, microalgae, and protozoa, in the water column. Fish and shrimps constantly eat this microbial biomass, which changes the microbial environment in the rearing water and the gut microbiome. This mechanism protects against infections in a number of ways. One way it does this is through competitive exclusion, where beneficial heterotrophic bacteria in biofloc compete for nutrients and space, making it tougher for harmful pathogens like *Vibrio* spp. to settle in [8,18]. Secondly, this occurs through antagonism, whereby numerous biofloc-associated taxa generate antimicrobial compounds (such as bacteriocins, organic acids, and enzymes) or participate in quorum quenching, thereby directly inhibiting pathogen growth and virulence signaling [5]. Thirdly, it transpires through microbiome maturation, wherein ongoing exposure to a complex environmental microbiome fosters the establishment of a more stable gut community, enhancing barrier function and digestion; this correlates with elevated expression of pattern-recognition receptors and immune genes in shrimp [12,44]. All of these effects work together to minimize the number of pathogens in the environment and make it less likely that a pathogen can successfully colonize a host. This type of effect is frequently called colonization resistance.

### Activation of innate immune responses

Shrimp possess no adaptive immunity; hence, the control of innate effectors is essential. Exposure to biofloc has consistently been associated with the enhancement of essential innate immune indicators, including hemocyte-mediated responses. Research indicates that shrimp reared in biofloc systems often exhibit elevated total hemocyte counts, enhanced phagocytic activity, and increased respiratory burst; while these responses are largely correlative, emerging evidence suggests that microbial-associated molecular patterns (MAMPs) and biofloc-derived microbial biomass may directly stimulate innate immune pathways [45,46]. These cellular alterations facilitate enhanced rapid elimination of invading microorganisms. Additionally, humoral effectors like phenoloxidase activity, lysozyme levels, and antimicrobial peptide gene expression (e.g., crustins, penaeidins) are frequently elevated in biofloc-cultivated prawns, indicating an enhanced humoral defensive preparedness [23,45]. Furthermore, immune priming occurs through the incorporation of MAMPs. The incorporation of microbial-associated molecular patterns (MAMPs), including peptidoglycan, lipoteichoic acids, and LPS, found in biofloc, serves as natural immunostimulants. This process primes hemocytes and innate signaling pathways (such as the prophenoloxidase cascade and Toll-like signaling), consequently reducing the activation threshold when faced with pathogens [5,43]. These responses are commonly assessed using functional assays (phagocytosis, NBT reduction), enzymatic assays (phenoloxidase, lysozyme), and gene expression analysis (RT-qPCR of immune genes). Hemocytes are the primary effector cells of innate immunity in all crustaceans, and reports of hyaline, semi-granular, and granular hemocytes have been made in prawns, crabs, and crayfish. Research on freshwater prawns and crabs (such as *Scylla* spp.) shows immunological characteristics that are similar to those seen in *L. vannamei*, such as nodulation, encapsulation, and respiratory burst activity in response to microbial assault [47].

### Antioxidant defense enhancement

Oxidative stress (higher ROS) caused by intensive cultivation conditions damages immune cells and general health. Biofloc systems seem to reduce oxidative stress in several ways, including through the antioxidants they provide in food. When consumed, biofloc particles that are high in carotenoids, vitamins C and E, and other antioxidants can strengthen the host's antioxidant capacity (SOD, CAT, and GPx activities) [44,45]. Additionally, it increased water quality, which decreased chronic stress. Heterotrophic microorganisms that effectively assimilate nitrogen reduce variations in ammonia and nitrite, which in turn lessens chronic physiological stress that would otherwise impair immunological functioning [17]. Moreover, the upregulation of antioxidant enzymes, evidenced by empirical investigations indicating elevated activity of SOD, catalase, and glutathione-related enzymes in biofloc-reared shrimps, is associated with reduced lipid peroxidation and enhanced hemocyte viability under stress [48]. Enhanced antioxidant defense systems help protect hemocytes and other immune-related tissues from oxidative damage generated during respiratory burst activity and environmental stress, thereby supporting sustained immune function in shrimp [49].

### Bioactive metabolites and immunostimulation

Biofloc microbial consortia generate various metabolites that can functionally influence prawn physiology and immunity. Short-chain fatty acids and other microbial compounds interact with gut epithelial cells through G-protein-coupled receptors, modulating mucus secretion and antimicrobial peptide expression. Exopolysaccharides generated by biofloc bacteria may function as adjuvant-like molecules, promoting hemocyte activation and the expression of antimicrobial peptides [5]. By promoting nutrient availability and digestion, the enzymes and digestive factors, such as lipases and proteases, included in biofloc can indirectly boost immunological competence by enhancing nutritional status [12]. Although numerous bioactive compounds are discovered in vitro, proving causation in vivo necessitates integrated metabolomic and functional studies; however, increasing evidence indicates a contributory role for these microbial metabolites in immunostimulation.

### Antiviral immune mechanisms in shrimp

Viral infections, particularly those produced by white spot syndrome virus (WSSV) and other DNA and RNA viruses, continue to be the most destructive limitations on prawn farming. Shrimp, unlike vertebrates, lack adaptive immunity and so rely solely on innate immune processes to identify, inhibit, and eradicate viral infections. Among these, RNA interference (RNAi), apoptosis, and conserved immunological signaling pathways form the primary antiviral defense system in penaeid shrimp.

RNA interference is largely regarded as the primary antiviral mechanism in crustaceans. During viral infection, Dicer enzymes break double-stranded RNA intermediates produced during viral replication into small interfering RNAs (siRNAs), which are then loaded into the RNA-induced silencing complex (RISC). This complex, guided by Argonaute proteins, targets and cleaves complementary viral RNA, reducing viral replication and propagation [[50], [51], [52]]. Functional investigations in *L. vannamei* have shown that silencing Dicer or Argonaute genes greatly enhances WSSV susceptibility, supporting RNAi's important antiviral role in prawns [53]. Apoptosis is another important way that shrimp fight viruses. It stops viruses from spreading by killing infected host cells. WSSV infection triggers programmed cell death in hemocytes and epithelial tissues, thereby restricting viral spread during the initial phases of infection [54,55]. On the other hand, WSSV has developed several anti-apoptotic genes to fight this reaction, which shows how important apoptosis is in the interactions between hosts and viruses [56]. Modulation of apoptotic pathways is a crucial factor influencing prawn survival during viral challenges.

Alongside RNA interference and apoptosis, other conserved innate immune signaling pathways play a role in the antiviral defense. The Toll, IMD, JAK/STAT, and MAPK pathways govern the transcription of antiviral and immune-associated genes, such as interferon-like factors, antimicrobial peptides, lectins, and pattern recognition receptors [27,57]. Following WSSV infection, components of these pathways are swiftly elevated, and experimental alterations in Toll and JAK/STAT signaling have been demonstrated to greatly influence viral loads and host survival [58]. These pathways have significant interaction with oxidative stress and metabolic signaling networks, highlighting the interconnectedness of antiviral immunity in prawns. Emerging research suggests that culture settings, such as biofloc-based systems, can alter antiviral competence by priming innate immune pathways, increasing antioxidant capacity, and modifying hemocyte activity. Improved redox balance and microbial-associated molecular pattern stimulation under biofloc circumstances may thus indirectly improve RNAi efficiency, apoptotic control, and antiviral signaling responsiveness, contributing to better resistance to viral infections.

### Disease resistance outcomes

The mechanistic alterations outlined previously result in functional advantages during pathogen exposure. It increased survivability, since several challenge studies indicate that biofloc-reared shrimp have greater survival rates when exposed to *Vibrio* spp., *WSSV*, or other pathogens than conventionally reared controls [45,59]. Additionally, pathogen loads and virulence decreased. Environmental factors that inhibit opportunistic bacteria, along with enhanced host defenses, contribute to decreased pathogen proliferation and reduced disease severity [8]. Furthermore, the treatment improved recuperation and resilience. Improved antioxidant and immunological health results in faster recovery from sublethal infections and fewer subsequent consequences. System variables (C:N ratio, carbon supply, solids content), host species, life stage, and biofloc management consistency all have a substantial impact on disease resistance [5,17]. Consequently, whereas BFT offers a multifaceted improvement in prawn immunity, its efficacy is contingent upon sufficient operational management.

### Biofloc shrimp immune responses: molecular and omics insights

Recent advancements in high-throughput transcriptome and metabolomic analysis have significantly improved our comprehension of prawn immune responses in biofloc and pathogen exposure scenarios. Comparative transcriptome analyses of *L. vannamei* cultivated in biofloc technology systems demonstrated significant differential expression of genes related to cellular stress response, immune recognition, and pathogen-responsive pathways, including those implicated in pathogen recognition, signal transduction, and stress alleviation. This indicates that biofloc conditions can influence host immune gene networks at the molecular level [60,61]. Integrated transcriptomic and metabolomic analyses after the White Spot Syndrome virus (WSSV) challenge revealed synchronized alterations in immune-related gene expression and metabolite profiles in the hepatopancreas, underscoring the critical functions of antioxidant systems, apoptosis regulation, and immune effectors during viral infection [60]. These investigations offer mechanistic proof that biofloc-mediated molecular-level immune and metabolic pathway regulation contributes to shrimp's increased immunological resilience. Additionally, transcriptomic profiling has shown that early WSSV infection activates neuroendocrine immune signaling pathways in hemocytes, highlighting the intricate interactions between immune regulation and other physiological systems [62]. When taken as a whole, these omics-based studies highlight the ways in which pathogen exposure and biofloc circumstances trigger particular molecular signatures in immune and stress pathways, bolstering the mechanistic basis of our analysis.

## Environmental and operational factors affecting immune benefits of biofloc technology in shrimp

The immunological benefits of BFT in shrimp aquaculture are significantly affected by environmental and operational factors that regulate microbial dynamics, nutrient cycling, and the general stability of the system. Biofloc can help with microbes and nutrients, but its ability to boost the immune system depends on how well the system is managed. The C:N ratio, the type of carbon source, the density and composition of the biofloc, the quality of the water, and the number of shrimps in the tank are all important aspects Table 2.

**Table 2:** Environmental and operational factors influencing immune benefits of biofloc technology (BFT) in shrimp.

Factor	Optimal Range	Immune-Related Effects	Mechanistic Explanation	Refs.
Carbon-to-Nitrogen (C:N) Ratio	15:1–20:1	↓ Ammonia/nitrite toxicity; ↑ Hemocyte count, phenoloxidase (PO) activity	Promotes heterotrophic bacterial assimilation of nitrogen; reduces toxic nitrogen stress and enhances microbial protein source	[8,17]
Carbon Source Type	Complex carbohydrates (tapioca, wheat flour) preferred over simple sugars	↑ Lysozyme activity, ↑ PO activity, ↑ survival after challenge	Supports stable microbial growth and beneficial bacteria (e.g., *Bacillus, Lactobacillus*) leading to immunostimulation	[5,45,46]
Biofloc Density & Composition	150–400 mg/L TSS; balanced microbial consortium	↑ Total hemocyte count (THC), ↑ phagocytic activity; ↓ oxidative stress	Moderate floc levels act as immunonutrients and provide competitive exclusion of pathogens	[12]
Water Quality	DO > 5 mg/L; pH 7–8; temp. 28–32°C	Stable immunity, reduced oxidative stress	Supports nitrification and microbial balance; optimal for enzyme activity and hemocyte metabolism	[10,17]
Stocking Density	Moderate densities (100–300 shrimp/m³)	↓ Stress-induced immune suppression; ↑ disease resistance	Prevents competition and stress hormone (CHH) elevation that impair immune responses	[43]
Feeding Regime	20–30% reduction in commercial feed in BFT	↑ Digestive enzyme activity; ↑ antioxidant enzymes (SOD, CAT)	Biofloc provides microbial protein and vitamins enhancing immune metabolism	[12,46]
Aeration & Mixing	Continuous and uniform	Prevents hypoxia and floc sedimentation	Maintains microbial diversity and suppresses opportunistic pathogens	[5]
System Stability	Long-term microbial balance	Sustained immune enhancement and growth	Stable microbial network prevents dysbiosis and stress	[43,69]

### Carbon-to-nitrogen (C: N) ratio

The most significant aspect of controlling a biofloc system is the C:N ratio, which regulates the balance of autotrophic and heterotrophic bacteria activity. Keeping the right C:N ratio, usually between 15:1 and 20:1, helps favorable heterotrophic bacteria grow. These bacteria take in inorganic nitrogen (ammonia, nitrite) and turn it into microbial biomass [63]. Reduced toxic nitrogen stress and decreased ammonia and nitrite levels are two important immunological benefits that these bacteria offer in optimal ratios. These bacteria also minimize physiological stress that inhibits prawn immune processes, such as hemocyte activity and phenoloxidase response [17]. It improved the quality of microbial feed, where heterotrophic microbial aggregates are nutritional supplements that are high in proteins, lipids, and vitamins that can help the immune system work better [8]. However, very high or very low C:N ratios can throw off the balance of microbes, which can cause oxygen deprivation, unstable pH, or too much organic loading, all of which might hurt the immune system of shrimp [64].

### Type of carbon source

The kind of carbon source has a big effect on the makeup of the biofloc microbial population and the profiles of its metabolites. These changes then affect the health and immunity of the prawn. Simple sugars like glucose and molasses help bacteria grow quickly, but they could also cause floc to develop in an unstable way. Furthermore, complex carbohydrates like starch and cellulose-based sources help microbes develop more slowly and create a stable floc with better nutrition [45]. In comparison to simple sugar systems, studies have demonstrated that prawns raised in systems supplemented with carbohydrate sources like tapioca or wheat flour have higher levels of lysozyme, phenoloxidase activity, total hemocyte counts, and improved immunological indicators [45,46]. The kind of carbon source also affects the microbial community, changing the number of positive bacteria like *Bacillus* and *Lactobacillus* spp. that help the gut and boost the immune system [5].

### Biofloc density and microbial composition

Shrimp physiological and immunological responses are strongly impacted by the amount and caliber of biofloc particles. The best microbial feed replenishment and water quality enhancement are achieved with a moderate floc density (150–400 mg/L TSS) [12]. On the other hand, excessive floc formation can counteract immunological benefits by causing oxidative stress, elevated ammonia, and hypoxia [43]. The composition of microorganisms within biofloc plays a crucial role in defining its functional characteristics. Systems characterized by *Bacillus, Nitrosomonas*, and lactic acid bacteria show improved immune parameters and increased resistance to pathogens [8]. On the other hand, transitions towards opportunistic *Vibrio species* due to inadequate management or high organic loading can result in disease outbreaks [48].

### Water quality parameters

To keep steady physicochemical conditions, biofloc systems need constant aeration and balanced microbial metabolism. Important factors like dissolved oxygen (DO), pH, temperature, and alkalinity have a big effect on both shrimp immune responses and microbial activity. For both heterotrophic bacteria to break down food and for oxidative immune responses such as the respiratory burst in hemocytes, the DO levels need to be higher than 5 mg/L [63]. The steady pH (7.0–8.0) and enough alkalinity (>100 mg/L as CaCO₃) help nitrification and stop acid stress, which would otherwise slow down immune enzyme activity [17]. Temperature has an effect on both the metabolism of shrimp and the kinetics of microbes. If the temperature goes outside of the ideal range (28–32°C), it can weaken the immune system and make biofloc less effective [46]. Keeping these factors in the right range keeps the advantageous microbial consortia active and the shrimp's bodies steady.

### Stocking density and feeding regime

Higher stocking densities are made possible by biofloc systems because of enhanced water quality and microbial recycling; however, too high a density can result in oxygen competition, nutrient limitation, and increased stress hormones like crustacean hyperglycemic hormone, all of which impair immune function [43]. In well-managed biofloc systems, particularly during the nursery and early grow-out phases, partial feed reduction has been reported as feasible because biofloc-derived microbial biomass can contribute supplemental protein, vitamins, and bioactive compounds. However, reductions commonly reported in the range of approximately 20–30% should be regarded as system- and phase-dependent guidelines rather than fixed values, and must be carefully adjusted to shrimp size, culture stage, and floc stability to enhance immune resilience without promoting excessive organic matter accumulation [12].

### System management and operational stability

Last but not least, to keep the functional balance of a biofloc system intact, it is necessary to maintain regular aeration, mixing, and solid removal efforts. It is possible for the microbial population to swiftly change towards pathogenic dominance, which would negate the benefits of the immune system, if there are fluctuations in aeration or biofloc collapse [5]. Consequently, a prerequisite for prolonged immunological improvement is the maintenance of long-term stability in the structure of the microbial population and the cycling of nutrients [15].

Biofloc-based culture systems work best when they are carefully managed and constantly monitored. This is because biofloc systems are more like dense, microbially driven ecosystems than simple rearing units. One of the most important things managers need to do is keep the water mixing and aeration robust and steady. Not only do high aeration rates help prawns breathe, but they also meet the high oxygen needs of heterotrophic and nitrifying microbial communities and keep biofloc particles in suspension [43]. Inadequate mixing can disrupt microbial activities and endanger shrimp health by encouraging floc sedimentation, localized hypoxia, and uneven nutrient delivery. Venturi injectors, air diffusers, and paddlewheels are frequently used to guarantee uniform circulation and stop anaerobic zones from forming [14].

Controlling suspended solids is another key operational difficulty. Excessive solids (> 500 mg/L TSS) can cause gill irritation, decreased respiration, reduced feed intake, and increased microbial oxygen use, while moderate biofloc concentrations improve nutrient recycling and offer supplemental nutrition [65]. Short-term aeration loss can induce mass mortality due to the combined respiratory needs of prawns and microbial biomass. Similarly, corrective solutions, such as quick carbon-source modification, partial sludge removal, alkalinity correction, and temporary feed restriction, are frequently used to stabilize deteriorating water quality conditions [14]. Microbial instability is a major biological weakness of biofloc systems, in addition to physicochemical concerns. Bioflocs usually support helpful and competitive microbial groups, but if the C:N ratio, solids loads, or organic inputs are not managed properly, they may favor bacteria that are opportunistic or could be harmful, such as *Vibrio* spp [44]. Disease outbreaks, weakened immunity, and weakened system resilience have all been linked to shifts towards opportunistic taxa's dominance [18]. Maintaining microbiological balance through controlled carbon dosing, solids regulation, and steady ambient factors is crucial for both system production and animal health.

These findings collectively underscore the necessity for rigorous technical supervision of biofloc technology. Biofloc systems offer significant benefits for nutrient recycling, water quality stabilization, and the immunological conditioning of prawns when effectively controlled. Biofloc systems are still at risk of rapid physicochemical and microbiological destabilization without strong aeration infrastructure, solids management, and real-time monitoring. This shows how important it is to have skilled staff and clear operational standards.

## Comparison between biofloc and conventional systems in shrimp culture

Over the past twenty years, shrimp farming has grown quickly, which has led to a greater need for sustainable production techniques that boost growth while lowering their impact on the environment. There are two main types of systems that are commonly used; conventional systems (clear-water or flow-through) and the newer biofloc technology (BFT). These systems are entirely unique when it comes to how they handle water, how microbes live, how they feed, and how productive they are overall.

Conventional shrimp farming systems depend on constant or periodic water exchange to keep the water clean and get rid of harmful metabolic wastes like ammonia and nitrite. These systems are common, but they often release a lot of effluent, which can cause nutrient buildup and damage to nearby aquatic ecosystems [66]. Additionally, traditional systems rely significantly on formulated feeds, which means that feeding expenditures are one of the major costs of prawn farming [67]. Microbial aggregates are absent in the water, which means that nutritional recycling is limited and feed conversion efficiency is low.

Biofloc technology, on the other hand, combines controlling water quality with adding extra nutrients by creating dense communities of microbes. Heterotrophic bacteria incorporate inorganic nitrogen into microbial biomass by keeping a high carbon-to-nitrogen (C:N) ratio, which is commonly done by adding carbohydrates [10]. This procedure lowers harmful nitrogen levels. These microbial flocs are a natural supply of protein that is already present in the environment. They help animals use their food better and make them less dependent on commercial feeds. Shrimp cultivated in biofloc technology systems often exhibit enhanced growth performance, improved feed conversion efficiency, and improved immunological responses, which are attributed in part to the continuous availability and consumption of beneficial microbial biomass [5,43].

Biofloc systems demonstrate enhanced environmental sustainability by necessitating minimal water exchange, efficiently transforming what would typically be waste in traditional systems into extra biomass [68]. Minimizing water usage contributes to limiting the spread of pathogens and reduces the release of nutrient-rich effluents. Nonetheless, the system requires a greater energy input to sustain ongoing aeration and mixing, which could lead to operational expenses that are potentially elevated compared to traditional systems [14]. Moreover, proficient personnel are essential for overseeing water quality in BFT, particularly in maintaining stable floc volume, alkalinity, and C:N ratio.

In general, BFT has clear benefits for recycling nutrients, using less water, and making prawns grow better. However, traditional systems are still easier to use, and they use less energy. The size of the farm, how well the manager can handle things, and how much it will cost are all factors that affect the choice between these two methods. Producers who want to maximize their work while being eco-friendly may find that hybrid or semi-biofloc systems are a good way to do it.

## Knowledge gaps and future directions

There is a lot of proof that biofloc systems can help *L. vannamei* grow faster and boost short-term immunological markers. However, there are still some big gaps in our understanding and some critical constraints that make it impossible to make solid mechanistic interpretations and practical suggestions. Here, we delineate the primary issues identified in the review and propose crucial areas for additional research.

### System variability and lack of reproducibility

A significant drawback in BFT investigations is the considerable diversity influenced by system design, management techniques, and host-related factors. Immunological outcomes are significantly affected by biofloc composition (heterotrophic versus nitrifying systems), carbon supply, carbon-to-nitrogen ratio, stocking density, salinity, aeration intensity, and culture time. Despite the application of same techniques, microbial populations and host responses may vary significantly across different facilities and experiments [69,70]. Additionally, inter- and intra-species variations, particularly strain-specific responses in *L. vannamei*, demonstrate that host genetics and developmental stages influence biofloc-immune interactions.

**Future direction:** The creation of comparable and standardized experimental frameworks, together with the thorough reporting of system parameters (C:N ratio, carbon supply, floc volume, microbial biomass, aeration regime, and solids load), is necessary for advancement in this discipline. Strong identification of the causative biofloc components would be possible with coordinated multi-site trials and reference system designs, which would significantly increase reproducibility.

### Predominance of short-term studies

The majority of studies on biofloc-mediated immunomodulation are laboratory-based and short-term and concentrate on proximal immunological markers like lysozyme levels, respiratory burst, phenoloxidase activity, and total hemocyte count. However, there is still little long-term data connecting BFT to enhanced survival in commercial settings, decreased illness incidence, and persistent immune resilience [71,72].

**Future direction:** There is an urgent need for multi-cycle, farm-scale research that combines immunological profiling with clinically relevant outcomes such as pathogen prevalence, cumulative mortality, growth-adjusted survival, and production stability. Such trials should include natural or controlled challenge testing and extended monitoring to assess immunological persistence or immune priming-like effects.

### Inconsistent immune markers and protocols

Various non-uniform endpoints, such as cellular assays, enzyme activities, oxidative stress indicators, gene expression assessments, and challenge tests, are used in current prawn immunology investigations. These endpoints are frequently assessed using various techniques and sample schedules. Meta-analysis and cross-study synthesis are highly constrained by this lack of standardization [69].

**Future direction:** The field would benefit from the development of consensus immunological marker panels customized to BFT systems, which would include hemocyte subpopulations, antimicrobial peptide expression, antioxidant enzymes, and functional disease resistance metrics. Harmonized sample points (baseline, mid-culture, and post-challenge) and interlaboratory validation techniques would significantly improve comparison power.

### Limited mechanistic resolution

While transcriptional and enzymatic alterations have been documented, the molecular pathways connecting biofloc-derived microbial products to host immunological signals are still inadequately elucidated. The functions of MAMPs, microbial metabolites, and dietary biofloc components in the activation of pathways like Toll, IMD, JAK/STAT, and prophenoloxidase cascades remain inadequately characterized [70,71].

**Future direction:** Future studies should focus on integrated multi-omics techniques that combine host transcriptomics and proteomics with floc metagenomics and metabolomics. Mechanistic mapping of the biofloc–immune interface will be made possible by these datasets, which are backed by functional validation (e.g., MAMP exposure assays, pathway inhibition studies, gnotobiotic or defined-community systems).

### Poorly resolved microbial functionality and stability

Most biofloc studies offer descriptive microbial profiles at a coarse taxonomic resolution, with insufficient evaluation of functional features, ecological relationships, or long-term community stability. The roles of bacteriophages, protozoa, and opportunistic bacteria, along with the effects of environmental disturbances, are mostly unexamined [71].

**Future direction:** In the future, microbial pathways involved in vitamin biosynthesis, short-chain fatty acid production, antimicrobial compound secretion, and redox regulation should be identified by combining functional metagenomics, metatranscriptomics, and metabolomics. To identify microbial profiles linked to long-lasting immunological advantages, disturbance-recovery tests and resilience testing will be crucial.

### Limited integration of immunology with operational and economic realities

BFT offers a strong advantage in immune improvement, but it rarely combines immunological results with economic viability, energy needs, labor requirements, and production dangers. Immunological benefits by themselves might not be sufficient to support widespread adoption in the absence of a techno-economic backdrop [70].

**Future direction:** Translational research models that link immune performance to cost-benefit assessments, energy efficiency, system stability, and regional disease ecology should be used in future investigations. For industry adoption, immunological integration with production modelling will be essential.

## Conclusion

Biofloc technology signifies a significant advancement in shrimp aquaculture, providing concurrent enhancements in environmental sustainability, nutrient recovery, and host immunological resilience. Mechanistic evidence from various experimental systems indicates that BFT improves prawn immunity through a network of interconnected pathways. These include the modulation of gut and water-column microbiota, the activation of innate immune effectors, the enhancement of antioxidant defenses, and the provision of immunostimulatory microbial metabolites. These responses work together to enhance resistance to pathogens and decrease vulnerability to diseases that limit production. Nonetheless, the immune advantages of BFT are heavily dependent on system setup, carbon management, microbial makeup, and operational consistency, leading to variability among studies and hindering the consistent application of research outcomes. While there has been notable advancement, significant obstacles continue to persist. The absence of standardized immune assays, the restricted molecular comprehension of host–microbe interactions, and the inadequacy of field-scale datasets impede the advancement of predictive models and the formulation of evidence-based operational guidelines. With the escalation of disease threats and the rise in climate variability, the importance of BFT in fostering strong and resilient prawn production systems is increasingly vital. Future research that integrates multi-omics technologies, immune-targeted probiotics, biofloc–vaccine synergies, and climate-adaptive BFT designs will be essential for transforming mechanistic insights into scalable, precision-managed aquaculture solutions. In conclusion, transitioning biofloc technology from practical experience to a framework grounded in mechanistic understanding and immune-optimized strategies will improve the sustainability, productivity, and biosecurity of crustacean aquaculture worldwide.

## Declarations

### Data availability statement

This review article utilized publicly available and cited data sources, including scholarly articles, books, reports, and online repositories. No new data were generated as part of this review. All sources referenced in this article are appropriately cited in the references section.

### Ethics approval

Not applicable.

### Funding

No funding.

## CRediT authorship contribution statement

**Yusuf Jibril Habib:** Writing – review & editing, Writing – original draft, Visualization, Validation, Supervision, Software, Resources, Methodology, Investigation, Conceptualization. **Shengkang Li:** Writing – review & editing, Visualization, Validation, Supervision, Software, Methodology, Investigation. **Mayada Alhoshy:** Writing – review & editing, Visualization, Validation, Software, Methodology, Investigation. **Islam I. Teiba:** Writing – review & editing, Visualization, Validation, Software, Methodology, Investigation. **Xianyuan Zheng:** Writing – review & editing, Visualization, Validation, Software, Methodology, Investigation. **Mohammed F. El Basuini:** Writing – review & editing, Writing – original draft, Visualization, Validation, Software, Resources, Methodology, Investigation. **Akram Ismael Shehata:** Writing – review & editing, Writing – original draft, Visualization, Validation, Software, Methodology, Investigation, Conceptualization.

## Declaration of competing interest

The authors declare no conflict of interest.

## Data Availability

No data was used for the research described in the article.
